# Synthetic and Natural Agents Targeting Advanced Glycation End-Products for Skin Anti-Aging: A Comprehensive Review of Experimental and Clinical Studies

**DOI:** 10.3390/antiox14040498

**Published:** 2025-04-20

**Authors:** Joon Yong Choi, Nam Gyoung Ha, Weon Ju Lee, Yong Chool Boo

**Affiliations:** 1 Department of Biomedical Science, The Graduate School, Kyungpook National University, 680 Gukchaebosang-ro, Jung-gu, Daegu 41944, Republic of Korea; halo134679@knu.ac.kr; 2 BK21 Plus KNU Biomedical Convergence Program, Kyungpook National University, Daegu 41944, Republic of Korea; 3 Department of Dermatology, Kyungpook National University Hospital, Daegu 41944, Republic of Korea; mkolp0515@gmail.com (N.G.H.); weonju@knu.ac.kr (W.J.L.); 4 Bio-Medical Research Institute, Kyungpook National University Hospital, Daegu 41944, Republic of Korea; 5 Department of Dermatology, School of Medicine, Kyungpook National University, Daegu 41944, Republic of Korea; 6 Department of Molecular Medicine, School of Medicine, Kyungpook National University, Daegu 41944, Republic of Korea; 7 Cell and Matrix Research Institute, Kyungpook National University, Daegu 41944, Republic of Korea

**Keywords:** advanced glycation end-products, skin aging, anti-aging, antiglycation, antioxidant, cosmetics, dermatology, plant extract, phytochemical, glycation inhibitor

## Abstract

Advanced glycation end-products (AGEs) cause blood vessel damage and induce diabetic complications in various organs, such as the eyes, kidneys, nerves, and skin. As glycation stress causes aesthetic, physical, and functional changes in the skin, glycation-targeting skin anti-aging strategies are attracting attention in cosmetology and dermatology. The primary goal of this review is to understand the significance of glycation-induced skin aging and to examine the therapeutic potential of glycation-targeting strategies. This study covers experimental and clinical studies exploring various interventions to attenuate glycation-induced skin aging. Glycation stress decreases the viability of cells in culture media, the cell-mediated contraction of collagen lattices in reconstructed skin models, and the expression of fibrillin-1 at the dermo-epidermal junction in the skin explants. It also increases cross-links in tail tendon collagen in animals, prolonging its breakdown time. However, these changes are attenuated by several synthetic and natural agents. Animal and clinical studies have shown that dietary or topical administration of agents with antiglycation or antioxidant activity can attenuate changes in AGE levels (measured by skin autofluorescence) and skin aging parameters (e.g., skin color, wrinkles, elasticity, hydration, dermal density) induced by chronological aging, diabetes, high-carbohydrate diets, ultraviolet radiation, or oxidative stress. Therefore, the accumulating experimental and clinical evidence supports that dietary supplements or topical formulations containing one or more synthetic and natural antiglycation agents may help mitigate skin aging induced by AGEs.

## 1. Introduction

When we talk about the aging of the body, skin aging is often the first thing that comes to mind because it is easily visible to the naked eye [1,2]. Among the various changes that accompany skin aging, the aesthetic changes of skin appearance, such as wrinkles, sagging, color, and gloss, are of particular interest in cosmetology, and the physical and functional changes in skin barrier integrity, immune response, wound healing, and other physiological aspects are of significant importance in dermatology [3,4]. Skin aging is theoretically divided into intrinsic and extrinsic types based on the origin of the causal factors, but in reality, the boundaries are ambiguous due to many overlapping factors [5,6]. Innate genetic traits and chronological aging are included in intrinsic skin aging factors [5], while environmental factors, such as ultraviolet (UV) rays, air pollution, and dry climate, are included in extrinsic skin aging factors [7,8,9]. On the other hand, smoking, drinking alcohol, drug use, and dietary habits may be considered mixed skin aging factors because these behaviors introduce external substances into the body, which then undergo chemical changes due to internal metabolism or environmental influences [10,11]. Intrinsic, extrinsic, and mixed skin aging factors can all have negative effects on the appearance and function of the skin in general.

Dietary carbohydrates are digested in the body to produce monosaccharides, such as glucose, fructose, and galactose. These monosaccharides are metabolized into small molecules through the glycolytic pathway and the citric acid cycle, generating NADH and FADH_2_, which are oxidized in the electron transport chain of the mitochondria for ATP synthesis [12]. Thus, under normal physiological conditions, carbohydrates function as essential nutrients that provide metabolites and energy required for cell survival and function. However, under pathological conditions (e.g., persistent hyperglycemia due to diabetes), they participate in non-enzymatic condensation reactions with proteins, lipids, and nucleic acids, forming advanced glycation end-products (AGEs) [13,14]. Glycation reactions also increase according to chronological age, inactive lifestyle, the consumption of high-carbohydrate diets, and exposure to UV rays [15,16,17,18]. AGEs are considered to be one of the representative mixed skin aging factors that combine external sources, intrinsic pathological alterations, and environmental exposures [19,20].

Reducing sugars, such as aldoses (e.g., glucose) and ketoses (e.g., fructose), can form Schiff bases in which their carbonyl groups are linked to free amine groups of various substrates, such as proteins, lipids, and nucleic acids, via the Maillard pathway or the polyol pathway [21,22]. These adducts then form early glycation products, such as Amadori products (e.g., glucose-derived AGEs) and Heyns products (e.g., fructose-derived AGEs), through intramolecular rearrangements. These early glycation products undergo oxidative degradation, generating various forms of reactive aldehydes (e.g., glyceraldehyde, glycolaldehyde), dialdehydes (e.g., diacetyl, glyoxal, methylglyoxal), diketones (e.g., 1-deoxyglucosone, 3-deoxyglucosone), etc., which participate in the formation of various types of AGEs [23,24,25,26]. Glycation products include the early glycation products (not included in AGEs), such as fructosamine and furosine; the intermediate glycation products, such as *N*^ε^-(carboxymethyl)lysine (CML), *N*^ω^-(carboxymethyl)arginine (CMA), and *N*^ε^-(carboxyethyl)lysine (CEL); and the late glycation products, such as *N*^δ^-(5-hydro-5-methyl-4-imidazolon-2-yl)-ornithine [also called “methylglyoxal-derived hydroimidazolone 1 (MG-H1)”], argpyrimidine, pentosidine, and glucosepane. The receptor for advanced glycation end-products (RAGE) is highly expressed in the skin [27] and mediates several cell-signaling pathways involved in inflammation, fibrosis, melanin synthesis, etc. [28,29]. AGEs stimulate the production of reactive oxygen species (ROS) via NAD(P)H oxidase- or mitochondrion-dependent mechanisms, worsening diabetes and diabetic complications [14,30,31,32]. Glycation inhibitors, such as aminoguanidine (AGD), and antioxidants, such as *N*-acetylcysteine and glutathione, have been shown to attenuate the toxic effects of AGEs [33,34,35]. Different types of breakers of the preformed AGEs have been studied as a therapeutic approach [36,37,38,39].

There are previous review papers that provide an overview of the molecular mechanisms, pathogenesis, and inhibition strategies of glycation reactions [15] and a comprehensive discussion of the therapeutic potential of various natural compounds that inhibit the formation of AGEs [40]. There are also review papers that focus on the skin, examining the formation and effects of AGEs in the skin, methods for measuring skin AGE levels, and strategies to reduce skin glycation stress [19,41]. Despite the existence of such excellent review papers, there is a lack of a comprehensive review of the experimental and clinical evidence supporting or opposing the therapeutic potential of various interventions on glycation-induced skin aging.

The goal of this review is to understand the significance of glycation-induced skin aging and to explore treatment strategies for the mitigation of glycation-induced skin aging. We comprehensively examine the experimental and clinical studies on the relationship between glycation and skin aging and the various interventions with synthetic and natural agents. This review limits its scope to skin aging induced by glycation, excluding other specific skin diseases. It covers studies at various levels, namely, in vitro studies undertaken using proteins, cells, and reconstructed skin models; ex vivo studies using live skin explants; in vivo studies using animals; and clinical studies involving human subjects. We hope that this comprehensive review will help recognize the significance of skin aging induced by AGEs and develop treatment strategies against glycation-induced skin aging.

## 2. Methods

We searched the PubMed (https://pubmed.ncbi.nlm.nih.gov/, accessed on 1 March 2025), Web of Science (https://www.webofscience.com/, accessed on 1 March 2025), and Google Scholar (https://scholar.google.com/, accessed on 1 March 2025) databases for experimental and clinical studies on the topic of this narrative review using various keywords, such as ‘advanced glycation end-products’, ‘skin aging’, ‘antioxidants’, ‘aminoguanidine’, ‘in vitro’, ‘ex vivo’, ‘in vivo’, ‘animal’, ‘mice’, ‘rats’, ‘chicks’, ‘clinical’, ‘synthetic’, and ‘natural’, using Boolean search commands, such as ‘AND’ and ‘OR’. The selected articles were cited and discussed in the appropriate chapter(s) or section(s) according to the level of studies, such as in vitro, ex vivo, in vivo (animal), and clinical studies.

## 3. Significance of Glycation-Induced Skin Aging

### 3.1. Experimental Evidence

As summarized in Table 1, increases in AGE levels in blood, skin, and other tissues due to diabetes, aging, and high-carbohydrate diets have been observed in various animal models. In addition, changes in skin appearance and function have been observed as skin AGE levels increase.

In rats and mice with diabetes induced by the injection of streptozotocin, the levels of AGEs in various tissues, such as blood, skin, liver, and ears, and the glycation of proteins, such as collagen and histone, increased [42,44,46,48,52]. In diabetic animals, the skin permeability and microbial barrier were weakened [46], and inflammation around the wound was worsened [52]. Protein glycation and carbamylation were responsive to diabetes and chronic kidney diseases, respectively, and there was a mutual competition between these two different types of protein modifications [51].

In several animal models, AGE levels in various tissues, such as skin, brain, and aorta, and the glycation of proteins, such as collagen, tended to increase with age [24,25,47,50,53]. This trend differed between breeders and non-breeders in mole-rats: breeders had higher AGE levels but longer lifespans [47]. Between mouse strains, those with higher glycemic levels had higher AGE levels, higher transepidermal water loss (TEWL) rates, stronger inflammatory reactions, lower skin moisture content, and lower collagen content than the other strains, and these differences expanded with age [53]. In lamin B receptor-deficient *ic^J^/ic^J^* mice showing symptoms of premature aging (progeria), AGE levels in the heart and liver were higher than in the control animals, although the differences in skin tissue were not evident [49].

In animal models, high-sugar diets increased AGE levels in blood and skin, increased collagen glycation, decreased collagen solubility, and increased urinary lipid peroxidation product levels [43]. They also increased the expression levels of collagen type I, fibronectin 1, laminin-5, and tenascin C among extracellular matrix proteins and receptors, along with AGE levels in the skin and darkened skin color [54]. On the other hand, they increased sebum secretion and decreased TEWL associated with enhanced fatty acid synthesis [54]. Feeding a high-AGE diet increased the AGE levels in the skin and collagen in diabetic *db/db*^(+/+)^ mice and delayed skin wound healing [44]. The subcutaneous injection of glycated collagen caused apoptosis of fibroblasts [45].

### 3.2. Clinical Evidence

As summarized in Table 2, several clinical studies have shown that AGE levels in the blood or skin of human subjects increase due to diabetes, aging, UV irradiation, smoking, etc., and are correlated with skin color and function and the incidence and severity of other diseases or complications.

In clinical trials, HbA1c, one of the glycated forms of Hb, is usually analyzed in human blood samples, and the fingertip skin autofluorescence is often measured as an indicator of AGEs in the skin. In diabetic patients, both blood HbA1c levels and skin autofluorescence tended to increase [55,63] and showed a mutual correlation [59], but there were exceptions [48]. Considering the relatively short lifespan of erythrocytes, AGE levels in the skin may reflect a longer-term accumulation of glycation products than those of Hb [66,67]. In addition, the actual levels of AGEs measured in blood or skin were correlated with the incidence or severity of cognitive impairment, glaucoma, and other diseases, regardless of whether the patient was diagnosed with diabetes [48,62,64].

The skin AGE level increased with age, and it showed a correlation with changes in skin color and muscle strength [57,58]. Skin AGE levels increased due to exposure to UV rays, and oxidative damage to DNA and lipid peroxidation also occurred [60,65]. In diabetic patients, persistent hyperglycemia decreased skin moisture and sebum, but TEWL was reduced in the high HbA1c group, so the effects of AGEs on skin physiology and function may not be straightforward [56].

The AGE level increased while galectin-3 expression was reduced at the edge of the skin wound bed in both diabetic and non-diabetic patients, supporting that galectin-3 may function as a receptor for AGEs involved in AGE clearance, and its absence leads to the accumulation of AGEs [61,68].

## 4. Interventions for Glycation-Induced Skin Aging

### 4.1. In Vitro Cell-Free Studies

Table 3 summarizes the selected in vitro studies on the glycation of protein substrates and the effects of potential glycation inhibitors of synthetic and natural origins.

To induce glycation in vitro, a protein substrate and a glycating agent are reacted in an aqueous medium. Albumin, the major protein of blood, and collagen and elastin, the major proteins of the dermis, are frequently used as protein substrates, which provide useful experimental models for glycation reactions in blood and skin, respectively. Glycation agents used include reducing sugars, such as glucose, fructose, and ribose; aldehydes, such as glycolaldehyde and glyceraldehyde; dialdehydes, such as glyoxal and methylglyoxal; and diketones, such as dehydroascorbic acid. The pH of the medium is usually maintained at 7.4 and the temperature at 37 °C to match the biological conditions, but the pH and temperature may be adjusted to accelerate the reaction rate. The total reaction time is adjusted according to the reactivity of the reactants, from several days to several weeks. In the case of a relatively long reaction time, it is common to add a preservative such as NaN_3_ to prevent changes caused by microorganisms. To quantify AGEs produced through in vitro glycation reactions, fluorescence is usually measured (fluorimetry) [71], although absorbance is often measured (colorimetry) after the nitroblue tetrazolium (NBT) reaction or elastase digestion. In addition, enzyme-linked immunosorbent assays (ELISAs) or dot blots using antibodies immunoreactive to general AGEs or a specific type of AGEs (e.g., CML) are also performed. The increase in protein size resulting from the glycation reaction is confirmed by a decrease in protein mobility in sodium dodecyl sulfate-polyacrylamide gel electrophoresis (SDS-PAGE).

In vitro, the glycation reaction is performed to produce protein-AGE adducts (52, 35) or to discover and evaluate inhibitors of the glycation reaction (Table 2). In studies for the latter purpose, AGD or alagebrium (ALT-711) is often used as a positive control. These studies reported that various materials, such as gold nanoparticles [73]; mycosporine-like amino acids [82,87]; *N*-acetylhydroxyproline [95]; carnosine [80]; phenolic compounds, such as silibinin [76], rosmarinic acid [80], quercetin [81], gallic acid [86], methyl gallate [89], apigenin, chlorogenic acid [90], vanillic acid [92], and flavolignans [83]; alkaloids [85]; polysaccharides [88]; and extracts derived from *Manilkara* and *Argania* [75], *Silybum marianum* flower [76], *Akebia quinata* fruit [77], *Cecropia pachystachya* [81], back cumin (*Nigella sativa*) seed [84], hazelnut (*Corylus avellana*) skin [86], *Cirsium japonicum* flower [90], cranberry [93], and chlorella (*Parachlorella beijerinckii*) [91] significantly inhibited protein glycation reactions. These materials are thought to function as glycation inhibitors that competitively participate in glycation reactions or as antioxidants that inhibit a series of oxidative reactions involved in the production of AGEs.

### 4.2. In Vitro Studies Using Cultured Cells and Reconstructed Skin Models

Cultured cell models are used to study the pathological responses induced by glycation stress (Table 4). Given their relevance to skin aging, dermal fibroblasts are mainly used, although epidermal keratinocytes and other cell types are also occasionally used [52,97]. Cells are grown into a monolayer on the bottom of a culture plate or a 3-dimensional structure in a collagen gel (for a reconstructed dermis or skin model). Cells are treated with AGE_S,_ protein-AGE adducts, or different glycation inducers, such as glyoxal, methylglyoxal, H_2_O_2_, etc. These treatments are sometimes combined with UV irradiation.

When cells were exposed to AGEs, protein-AGE adducts, or conditions that increase AGEs (e.g., several glycating agents, t-butylhydroperoxide, H_2_O_2_, or UV radiation), cell senescence or death (apoptosis) increased [50,77,109,111]. ROS production increased [77,79], and antioxidant enzymes decreased [111], leading to increased oxidative stress and endoplasmic reticulum stress [50]. RAGE expression increased [79,110], and various cell signaling pathways were activated, resulting in the increased expression of inflammatory cytokines [79,109,111]. Gene expression of matrix metalloproteinases (MMPs) increased, whereas collagen gene expression decreased, resulting in the lowered collagen level [79,90,105,109,111]. These changes were further amplified by the combination of glycating agents and ultraviolet irradiation [96].

The glycation-induced changes in human dermal fibroblasts (HDFs) were reversed by various interventions, including AGD [109]; *N*-acetylcysteine [50]; carnosine [103,107]; supramolecular carnosine [110]; polyphenolic compounds, such as plantamajoside [79], resveratrol, oxyresveratrol, piceatannol [107], plant extracts derived from *Akebia quinata* fruit [77], and unripe *Carica papaya* fruit [109]; hydrolyzed fish collagen [104]; and the K formulation containing hyaluronan and collagen peptide [111]. In HaCaT keratinocytes, sunflower sprout extract inhibited AGE formation induced by UVA irradiation [106], and plantamajoside attenuated ROS production and the inflammatory response induced by bovine serum albumin (BSA)-AGE adducts and UVB irradiation [79]. *Pholiota nameko* polysaccharides and Djulis (*Chenopodium formosanum*) extract attenuated ROS production and enhanced the viability of Hs68 cells treated with methylglyoxal or CML [88,105]. Vanillic acid enhanced the viability of RAW264.7 cells exposed to methylglyoxal [92]. Hazelnut (*Corylus avellana*) skin extract and carnosine improved the vitality and phagocytic function of macrophages derived from THP-1 cells in removing senescent cells [94,103].

The antiglycation effects of *Jasminum sambac* cell extract were observed in an experimental model in which cultured cells were fixed with formalin and then treated with glyoxal to explore the production of AGEs [108]. Chemical changes due to glycation can be studied using dead dermis, which is prepared by killing the cells of the separated skin and removing the epidermis. UV irradiation caused the production of ROS and an increase in AGEs in the dead dermis, and these changes were alleviated by AGD and *Argania* extract [75]. Glyoxal treatment of mouse skin explants without cultivation caused the yellowing of skin color and increased TEWL, suggesting that glycation causes aesthetic and functional changes in the skin [97].

In a reconstructed dermis model in which HDFs were embedded in a three-dimensional collagen gel, the collagen gel contracted as the cells grew, whereas the change was inhibited in the glyoxal-treated collagen gel [91]. Chlorella (*Parachlorella beijerinckii*) extract, AGD, and *N*-acetylhydroxyproline inhibited the production of AGEs and recovered the cell-mediated collagen contraction [91,95].

The effects of collagen glycation have been studied in a reconstructed skin model with a stratified structure in which dermal keratinocytes are grown on top of a dermal-like structure after HDFs are grown in a collagen matrix with or without glycation [74,78,98,112]. Ribose-induced glycation of collagen inhibited the contraction of the collagen lattice, increasing the epidermal thickness and decreasing the dermal thickness of a reconstructed skin model [85,112]. Ribose increased CML levels and extended the suprabasal integrin β1 expression, whereas most of these changes were attenuated by AGD or blueberry extract [71]. In the skin model (Mimeskin^TM^, BASF BC, Lyon, France) reconstructed with ribose-treated collagen, the diameters of collagen fibers were reduced, and cell growth and layer formation were inhibited, but these changes were alleviated by AGD and *Davilla* extract with anti-glycation effects [75]. AGD or several alkaloids from *Ocotea Paranapiacabensis* inhibited the thinning of the dermal layer in skin models reconstructed using different glycated collagens [78,85].

In an endothelialized and innervated reconstructed skin model prepared from collagen-chitosan sponge seeded with HDFs, human umbilical vein endothelial cells (HUVECs), sensory neurons from the dorsal root ganglia of mouse embryos, and human epidermal keratinocytes, glyoxal increased CML and decreased the number of capillaries and the expression of angiogenesis markers, such as platelet endothelial cell adhesion molecule (PECAM) 1, loricrin, filaggrin, and Krüppel-like factor (KLF) 4, and these changes were attenuated by AGD but not alagebrium (ALT-711) [101].

In a full-thickness skin model (EpiDermFT, MatTek Life Sciences, Ashland, MA, USA), carnosine, resveratrol, oxyresveratrol, and piceatannol attenuated the cellular and structural changes induced by glycation stress [107].

### 4.3. Ex Vivo Studies

Human skin explants provide an ex vivo model that allows observation of the histological changes due to glycation stress imposed during the subsequent cultivation (Table 5).

Methylglyoxal increased the expression of CML during culture period, while decreasing the expression of fibrillin-1 protein [76,77,108], a component of microfibrils present in oxytalan fibers at the dermo-epidermal junction [116]. These changes were alleviated by AGD, carnosine, methyl gallate, and extracts of *Silybum marianum* flowers, *Akebia quinata* fruits, *Jasminum sambac* cells, and *Dunaliella salina* [76,77,89,108,113,114]. In skin specimens, glucose or glycolaldehyde treatment increased autofluorescence, which was reduced by incubation with *N*-acetylhydroxyproline [95] or deglycating enzymes, such as fructosamine 3-kinase and fructosyl-amino acid oxidase [115]. Fructosamine 3-kinase further improved the elasticity of hypertrophic scar tissue ex vivo [115].

### 4.4. In Vivo Animal Studies

Table 6 summarizes selected in vivo studies that explored the effects of various interventions on AGE accumulation and pathological changes induced by aging, diabetes, high-dose sugar administration, UV irradiation, etc.

In rats with streptozotocin-induced diabetes, AGD, rutin, and vanillic acid reduced the AGE levels in the skin [92,117]. Streptozotocin-induced diabetes in hairless mice increased skin AGE levels and wrinkles and decreased skin hydration and elasticity; these changes were attenuated by an AGE blocker containing goji berry, fig, and Korean mint extracts [135]. Red blood cell-immunoglobulin (Ig) G crosslinking and tail tendon collagen crosslinking increased in Lewis rats with streptozotocin-induced diabetes, whereas the oral administration of ALT-711, an AGE breaker, reduced these changes [36]. Topical lotion containing ALT-711 improved skin elasticity and hydration in Fischer 344 rats [36]. On the other hand, AGE-breakers, such as *N*-phenacylthiazolium and *N*-phenylacy-4,5-dimethylthiazolium halides, or pyridoxamine had no effects on the crosslinking of skin collagen and acid-solubility of tail collagen affected by streptozotocin-induced diabetes in rats [37].

In rats, mice, chicks, and monkeys, dietary restriction reduced the increase in the levels of AGEs, such as furosine, CML, and pentosidine, in the skin or skin collagen induced by chronological aging [26,119,120,122,128]. In Emory mice prone to age-related cataracts, dietary restriction reduced cataract grade and dermatological lesions and shortened the tail tendon breakdown time [118]. AGD, vitamins C and E, and green tea extracts had little or weak effects on skin AGE levels and tail tendon breakdown time in aging rats [126,127]. The increments in the AGE level due to aging and the effect of dietary restriction were smaller in the aorta and blood than in the skin [120]. The type of carbohydrates (cornstarch, sucrose, glucose, fructose, or a combination of glucose and fructose) in the diet had little effect on serum glycemic stress and AGE levels, whereas overall calorie restriction had significant effects in aging Fischer 344 rats [124].

In mice orally administered high-dose glucose, blood glucose levels were unchanged, but AGE levels in the subcutaneous tissue were elevated, microvessel diameters were reduced, and vascular lesions were increased; these changes were alleviated by drinking water containing AGD [121]. In mice administered high concentrations of galactose, blood AGE levels were elevated, and cognitive impairment was aggravated, which was alleviated by AGD [123]. In addition, the increase in skin AGE levels by galactose was accompanied by increased oxidative stress, decreased collagen expression and dermal thickness, and inhibition of microvascular development, and these skin aging-related changes were alleviated by adiposepderived stem cell injection [129,130]. *Lactobacillus fermentum* CQPC04-fermented soy milk and dapagliflozin (a medication used to treat type 2 diabetes) exhibited antioxidant, anti-inflammatory, and antiglycation effects in the skin and enhanced dermal collagen or hyaluronan in mice administered high galactose [132,136].

In UV-irradiated mice, the skin level of AGEs was reduced by *Schizonepeta tenuifolia* extract (containing rosmarinic acid) and idebenone-loaded nanoparticles [133,134]. In addition, *Schizonepeta tenuifolia* extract improved skin wrinkles, hydration, and the dermal levels of collagen and hyaluronan, and idebenone-loaded nanoparticles showed skin-whitening and antioxidant effects [133,134]. Mycosporine-like amino acid-containing emulsions enhanced the expression levels of antioxidant enzymes, such as SOD and CAT, in the ear skin irradiated with UV, although it had no significant effects on the skin AGE levels in DBA/2CrSlc mice [87].

In chicks, allopurinol injections reduced uric acid, which has antioxidant activity, causing oxidative stress, while hemin reduced uric acid, but both increased pentosidine in the skin and were relieved by dietary restriction [125].

### 4.5. Clinical Studies

Dietary and topical agents with antioxidant and antiglycation activities have been tested in clinical studies to address their effects on skin aging (Table 7).

The clinical effects of dietary supplements containing synthetic or natural substances with antioxidant and antiglycation activities on human skin aging parameters vary widely. In diabetic patients, dietary supplementation with vitamins E and C with antioxidant activity had no significant effect on skin AGE levels [137]. The oral intake of a capsule containing vitamins, amino acids, and carnosine with antiglycation activity improved skin surface parameters compared to the placebo control group [140]. An equol supplement containing *S*-equol and several other natural phenolic compounds did not improve skin aging parameters nor AGE levels, although it improved climactic symptoms in post-menopausal women [141]. Fish-derived collagen peptides reduced skin AGE levels and insulin resistance indices compared to the placebo control group [142]. Twelve weeks of intake of capsules containing rosemary extract reduced the levels of 4-hydroxynonenal-protein adducts and AGEs in the skin, supporting its antioxidant and antiglycation activity [144]. A green tea oral supplement had no significant effects on skin aging parameters even when its intake was combined with the topical application of a cream containing green tea extract enriched with (−)-epigallocatechin gallate and other phenolic compounds [138].

Several topical formulations have shown significant efficacy in improving human skin aging parameters. A topical formulation containing C-xylose and glycation-inhibitory blueberry extract mitigated skin aging symptoms, although the skin AGE level was not reduced significantly [139]. A cream containing glycation-inhibitory *Argania* plant extract, α-tocopheryl acetate, rutin, and ferulic acid lowered free radical production as measured by UVA-induced chemiluminescence [75]. An ampule containing hydrolyzed fish collagen (25% tripeptide) reduced skin AGE levels and improved periorbital and glabellar skin wrinkles, skin surface elasticity, and dermal density [104]. A gel cream containing *Dunaliella salina* extract rich in colorless carotenoids (phytoene and phytofluene) reduced skin AGE levels and inflammatory skin reactivity to histamine stimulation while improving skin aging parameters, including periocular wrinkles and red spots [114]. A lotion containing *Cirsium japonicum* flower extract also improved skin aging parameters [90].

A serum containing sunflower sprout extract exhibited antiglycation activity in cells and anti-inflammatory effects in an ex vivo model and improved facial skin conditions, such as radiance, smoothness, fine lines of crow’s feet, and overall eye appearance, in a human study [106]. An essence containing supramolecular carnosine with antiglycation activity reduced brown spots and the melanin index in the face and brightened the skin tone [110].

## 5. Discussion

### 5.1. Skin Anti-Aging Strategies Targeting Glycation

Glycation stress and oxidative stress are interconnected conditions with overlapping mechanisms of action, yet they also exhibit distinct effects. Oxidative stress due to an increase in prooxidants (e.g., ROS) and a decrease in antioxidants and antioxidant enzymes causes oxidative damage to proteins, lipids, and nucleic acids [145]. In contrast, glycation stress causes chemical modifications of proteins, lipids, and nucleic acids by sugars (glucose, fructose, and galactose), their metabolites, or their breakdown products [15]. On the protein side, protein carbonyls are formed via oxidation [24], and protein-AGE adducts are formed via glycation or glycoxidation [126], which are distinct from another type of protein modification, carbamylation [51]. Because of the mechanistic redundancy of glycation stress versus oxidative stress, in principle, many known antioxidants may be used in alleviating glycation stress and the associated skin aging [146,147,148]. Currently, targeting glycation with competitive glycation inhibitors or antioxidants is considered to be a practical and feasible skin anti-aging strategy [149,150].

**Figure 1:**
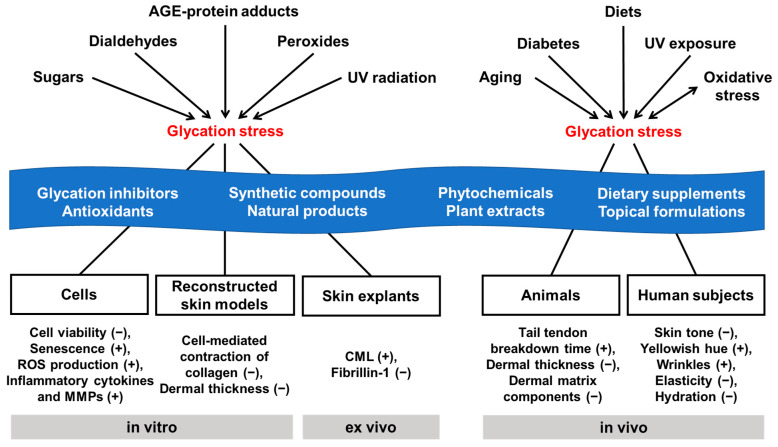
Causative factors for glycation stress-associated skin aging and interventions using synthetic and natural agents with antiglycation or antioxidant activity. Sharp arrows (↓) indicate stimulation. Plus and minus signs in parentheses indicate increases and decreases, respectively. Interventions are indicated with a blue background.

It is necessary to recognize that glycation stress may be crucial, especially in patients with diabetes, older people, and people who mainly eat carbohydrate-based meals. These people are advised to reduce high-carbohydrate diets, high-AGE diets, and UV exposure or to increase physical activity in addition to medical management [151,152,153]. It is clear that eliminating the cause of glycation stress through disease management and lifestyle modification is the primary option for glycation-induced skin aging. A secondary option may be the appropriate use of dietary supplements or topical formulations containing anti-glycation agents or antioxidants. Multiple levels of evidence from in vitro, ex vivo, in vivo, and clinical studies over the past 30 years support that glycation-induced skin aging can be mitigated by certain synthetic and natural agents with antiglycation and/or antioxidant activity (Figure 1).

In vitro experiments showed that several synthetic compounds, natural products, and plant extracts had anti-glycation activity (Table 3). In experiments using cultured cells, glycation stress increased ROS production, the gene expression of inflammatory cytokines, and cell death or senescence, which were suppressed by several synthetic and natural agents (Table 4). In reconstructed skin models prepared using glycated collagen, cell-mediated collagen contraction was impaired, and dermal thickness was reduced, whereas these changes were partially restored to normal by several synthetic and natural agents (Table 4). In ex vivo experiments using human skin explants, several natural and synthetic agents restored the expression of fibrillin-1 at the dermo-epidermal junction, reduced by glycation stress (Table 5).

In animal models, glycation stress due to diabetes, aging, high-carbohydrate diets, UV radiation, and oxidative stress increased AGE levels in the blood or skin and oxidative damage to blood or skin, decreased dermal thickness and the dermal contents of collagen and hyaluronan, and prolonged the tail tendon breakdown time (Table 1 and Table 6). Dietary restriction, oral and topical administration of synthetic or natural agents, and other interventions reversed these changes and alleviated glycation-induced dermal lesions or skin aging symptoms (Table 6). In clinical trials, several dietary supplements or topical formulations containing vitamins, amino acids, peptides, plant extracts, or phytochemicals reduced skin autofluorescence (or the AGE level), which was increased by diabetes, aging, high-carbohydrate diets, or UV irradiation (Table 2 and Table 7). They also improved skin aging parameters, such as skin tone, wrinkles, elasticity, and hydration, in human subjects (Table 7).

### 5.2. Synthetic and Natural Agents Targeting Glycation

There are several synthetic compounds that inhibit the formation of AGEs or decompose preformed AGEs, with studies supporting or questioning their therapeutic potential and safety. AGD has been used as a positive control substance in many studies and exhibits various biological effects but is not clinically used due to concerns about insufficient safety/efficacy balance or off-target effects [126,154]. Alagebrium (ALT-711), the first AGE-breaker, and later version compounds, such as *N*-phenacylthiazolium and *N*-phenylacy-4,5-dimethylthiazolium halides, also had similar shortcomings, limiting their applications [37,155].

Various other synthetic compounds, such as amino acids or derivatives (e.g., histidine, methionine, *N*-acetylcysteine, *N*-acetylhydroxyproline), peptides (e.g., carnosine, supramolecular carnosine), vitamins or their derivatives [e.g., vitamin B5 (pantothenic acid), pantethine, vitamin B6 (pyridoxine, pyridoxamine), vitamin C (ascorbic acid), vitamin E (α-tocopherol), α-tocopheryl acetate], and drugs (e.g., dapagliflozin), have been studied for application to glycation-induced skin aging, but their efficacy has not yet been well proven [137].

Among natural agents, phenolic compounds distributed in plants (e.g., rutin, quercetin, apigenin, rosmarinic acid, gallic acid, methyl gallate, vanillic acid, chlorogenic acid, ferulic acid, *S*-equol, resveratrol, oxyresveratrol, piceatannol, silibinin, plantamajoside) have been studied extensively for their antiglycation and skin anti-aging effects. Mycosporine-like amino acids derived from algae (e.g., *Agarophyton chilense*, *Pyropia plicata, Champia novaezelandiae*, *Bostrychia scorpioides*) or cyanobacteria (e.g., *Aphanothece halophytica*), flavonolignans derived from plants (e.g., *Silybum marianum*), alkaloids derived from plants (e.g., *Ocotea paranapiacabensis*), polysaccharides derived from mushrooms (e.g., *Pholiota nameko*) or bacteria (e.g., *Klebsiella pneumonia*, *Klebsiella planticola*), fish (e.g., *Pangasius hypophthalmus)*-derived collagen hydrolysates, and *Lactobacillus fermentum*-fermented soy milk have been studied for their applications in similar contexts.

Extracts from various plants have been studied with respect to glycation-induced skin aging, including *Akebia quinata* (Chocolate vine) fruit, *Argania spinosa* (Argan), *Agastache rugose* (Korean mint), *Camellia sinensis* (Green tea), *Carica papaya* (Papaya) fruit, *Cecropia pachystachya* (Ambay pumpwood) leaf, *Chenopodium formosanum* (Djulis), *Cirsium japonicum* (Japanese thistle) flower, *Corylus avellana* skin (Hazelnut), *Davilla rugosa* (Davilla), *Ficus carica* (Fig), *Helianthus annuus* (Sunflower) sprout, *Jasminum sambac* (Arabian jasmine) cell, *Lycium chinense* (Goji berry), *Manilkara multinervis* (African Manilkara), *Nigella sativa* (Black cumin) seed, *Rosmarinus officinalis* (Rosemary) leaf, *Schizonepeta tenuifolia* (Japanese catnip), *Silybum marianum* (Milk thistle) flower, and *Vaccinium angustifolium* (Blueberry). Additionally, microalgae, such as *Dunaliella salina* (Dunaliella) and *Parachlorella beijerinckii* (Chlorella), have also been studied for their antiglycation and anti-aging properties.

Although the therapeutic potential of many natural products on skin glycation and aging has been supported by previous studies, there have been exceptional observations as well [138,139]. In addition, since the experimental data of each natural product have been provided in individual studies, it is difficult to make a relative comparison between their efficacies. Furthermore, clinical trials to verify their skin anti-aging efficacy are mostly lacking.

### 5.3. Mechanistic Insights and Therapeutic Applications

Skin aging due to glycation stress can occur in both the epidermis and dermis [19,41]. Glycation stress increases the level of fluorescing yellowish AGEs in the epidermis and the cross-linking between the dermal matrix components, such as collagen, elastin, and hyaluronan. As a result, the skin color turns more yellowish and darker, the skin surface becomes rougher, and wrinkles increase and deepen. In addition, the dermis layer becomes thinner and harder, and elasticity decreases. Glycation stress can damage the skin barrier and increase TEWL rates [53,97,156], with some exceptional cases [54,56]. Therefore, skin aging due to glycation stress is recognizable by the aesthetic, physical, and functional changes of the skin.

**Figure 2:**
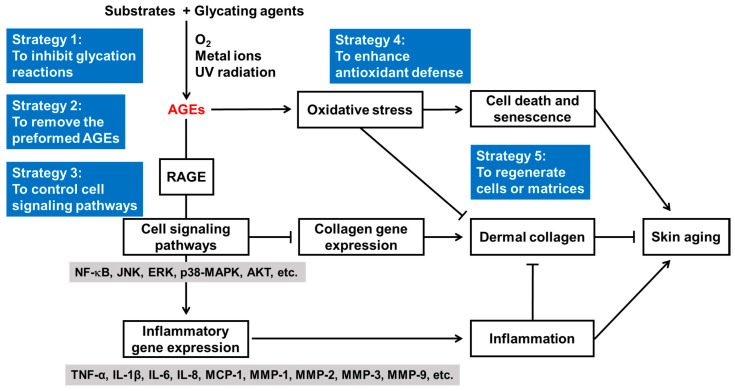
Potential therapeutic targets and treatment strategies against glycation-induced skin aging. Sharp (↓) and blunt (⊥) arrows indicate stimulation and suppression, respectively.

If we divide the process of glycation causing skin aging into two parts, the first step is the production of new AGEs through chemical reactions, and the second step is the occurrence of biological effects induced by existing AGEs (Figure 2). In the first step, when a glycation agent reacts with a substrate, the reaction is affected by oxygen, catalytic metals, UV rays, etc. [157,158]. In the second step, AGEs cause oxidative stress and stimulate RAGE-mediated cell signaling pathways, resulting in the upregulation of inflammatory cytokines and the downregulation of collagen. Overall, glycation stress leads to cell death or senescence, inflammation, and skin aging. Therefore, various treatment strategies against glycation-induced skin aging can be envisioned, such as strategy 1 to prevent the production of AGEs, strategy 2 to remove the preformed AGEs, strategy 3 to control RAGE-mediated cell signaling pathways leading to the upregulation of inflammatory gene expression and downregulation of collagen gene expression, strategy 4 to assist or enhance cellular antioxidant defense, and strategy 5 to regenerate cells and matrix environments.

For the successful clinical application of these strategies, the discovery and development of synthetic compounds, natural products, or other preparations suitable for each individual strategy must be a prerequisite. It is recommended that dietary supplements or topical formulations applied for human anti-aging purposes include at least one active component optimized for each strategy. Furthermore, it is also advantageous to apply multiple strategies in combination. Additionally, if possible, it may also be advantageous to apply oral supplements and topical formulations simultaneously. In any case, it is essential to check safety in advance. In clinical trials, the analysis of skin AGE levels along with various skin parameters should be conducted to verify whether the antiglycation intervention was performed properly. The level of AGEs in the skin can be conveniently quantified by measuring skin autofluorescence [66,143,159,160,161]. This method has the advantage of being noninvasive and minimally affected by the interference of other skin pigments such as melanin [162,163].

### 5.4. Future Tasks and Prospects

Developing technologies to implement the five therapeutic strategies to alleviate glycation-induced skin aging will be an important and necessary future task. Various glycation inhibitors for strategy 1 have been discovered in separate studies, making it difficult to know which one is better. An integrated study comparing their efficacy and safety with a set of criteria in the same experimental model is needed. For strategy 2, due to the questionable safety and efficacy of existing synthetic AGE-breakers [101,155], future studies are needed to develop alternative innovative therapies involving deglycating enzymes [115,164], oxidized protein hydrolases [165,166], or physical disruption of AGEs [167,168,169]. Additionally, we anticipate the development of drugs that promote the removal of AGEs by cell-mediated phagocytosis [103]. For strategy 3, although various synthetic and natural modulators are known to modulate the RAGE-mediated cell signaling pathways [170,171], many tasks remain to be implemented, including the assessment of safety, efficacy, pharmacodynamics, and pharmacokinetics in vivo. For strategy 4, various types of synthetic or natural antioxidants [172] and modulators of nuclear factor erythroid 2-related factor (NRF) 2 may be used to assist or enhance cellular antioxidant defense systems [173]. Future studies are needed to verify their efficacy in vivo before clinical application. Strategy 5 is the most challenging, but various attempts have already been made to help the regeneration of cells and matrix environments by injecting cells [129,130], matrix components [174], or skin boosters [175]. Developing advanced nanomaterials for the control of glycation stress is also an important future task for implementing these treatment strategies [73,134,176].

It is predicted that future skin anti-aging treatments will be led by targeted and tailored therapies that are supposed to vary by the specific cause of skin aging and the individual health condition of the subject. Such glycation-targeting treatments will be particularly important for patients who are more affected by glycation stress. In other words, the treatments will have a significant impact on skin aging only in subjects with a high skin AGE level. Therefore, for the best outcomes, the selection of the right treatment subjects is as important as the selection of the right treatment options. It is also important to measure skin AGE levels before and after treatments to check the quantitative changes due to the applied intervention. The data will be essential to claim the skin-anti-aging effects of a certain intervention targeting AGEs. The glycation-targeting technologies would have expanding applications in various skin inflammatory conditions (e.g., psoriasis, atopic dermatitis), metabolic complications (e.g., diabetic dermopathy, impaired wound healing), and age-related pathologies (e.g., elastosis, dyschromia).

## 6. Conclusions

Many pieces of experimental and clinical evidence support the significance of glycation-induced skin aging. Several synthetic and natural agents with antiglycation or antioxidant activity reduce skin AGE levels, oxidative damage, and skin aging symptoms caused by chronological aging, diabetes, high-carbohydrate diets, UV radiation, or oxidative stress. Therefore, it is suggested that glycation-targeting dietary supplements or topical formulations containing these agents may provide therapeutic options to mitigate glycation-induced skin aging.

## Figures and Tables

**Table 1:** Animal studies on the detection of advanced glycation end-products (AGEs) in the skin and observation of glycation-induced skin aging.

Model	Glycation Inducer	Findings	References
Sprague–Dawley rats	Diabetes induced by streptozotocin (70 mg kg^−1^, i.p.)	1. Increased glycated plasma proteins, glycated hemoglobin (Hb), and fructosamine. 2. The AGE levels of liver histones and skin collagen increased with the duration of diabetes and animal age.	Gugliucci and Bendayan, 1995 [42]
Sprague–Dawley rats	Glucose, sucrose, or fructose in drinking water (250 g L^−1^)	1. Blood glycated Hb levels and urine lipid peroxidation products were higher in fructose-fed rats. 2. Insoluble collagen and collagen-bound fluorescence were higher in fructose-fed rats.	Levi and Werman, 1998 [43]
*db/db*^(+/+)^ mice	High-AGE diets	1. Increased the levels of AGEs, such as *N*^ε^-(carboxymethyl)lysine (CML) and methylglyoxal derivatives, of skin proteins and collagen in diabetic mice. 2. Delayed skin wound closure in diabetic mice.	Peppa et al., 2003 [44]
CD1 mice	CML-collagen injection	1. Induced fibroblast apoptosis mediated by the receptor for advanced glycation end-products (RAGE) and caspases 3, 8, and 9. 2. Enhanced mRNA levels of pro-apoptotic genes.	Alikhani et al., 2005 [45]
Fatty OLETF rats and control LETO rats	Long-standing hyperglycemia	1. Fatty OLETF rats with diabetes or impaired glucose tolerance had higher serum AGE levels and epidermal RAGE levels. 2. Impaired skin barrier functions, including transdermal permeability and antimicrobial barriers.	Park et al., 2011 [46]
Ansell’s mole-rats (*Fukomys anselli*)	Age (1–19 years) and breeding status	1. Glucosepane, pentosidine, CML, and *N*^δ^-(5-hydro-5-methyl-4-imidazolon-2-yl)-ornithine (MG-H1) in insoluble skin collagen increased with age. 2. Glucosepane and CML were higher in breeders versus nonbreeders. 3. The pentosidine formation rate was lower in mole-rats than in other short-lived rodents.	Dammann et al., 2012 [47]
C57BL6/J mice	Age	1. Argpyrimidine and pentosidine, but not protein carbonyls, increased in the skin of old mice.	Nowotny and Grune, 2014 [24]
ddY mice	Diabetes induced by streptozotocin (150 mg kg^−1^, abdominal injection)	1. Increased blood glucose and fluorescent AGE levels in the auricle and decreased body weight.	Yamanaka et al., 2016 [48]
Lamin B receptor-deficient *ic^J^/ic^J^* mice (NMRI background)	Progeria (premature aging symptoms)	1. Pentosidine and argpyrimidine, but not CML, increased in the heart and liver (not skin) of *ic^J^/ic^J^* mice.	Hause et al., 2018 [49]
C57BL/6J mice	Age	1. Increased the levels of methylglyoxal-modified AGEs (argpyrimidine and MG-H1) in brain tissue and collagen.	Nowotny et al., 2018 [50]
ddY mice	20 weeks	1. The skin level of *N*^ω^-(carboxymethyl)arginine (CMA) was higher than CML, *N*^ε^-(carboxyethyl)lysine (CEL), and MG-H1.	Kinoshita et al., 2019 [25]
*db/db* [C57Bl/KsJ-*db/db*] mice and the corresponding controls (db^/+^)	Chronic kidney disease by nephrectomy or cyanate-supplemented water	1. Diabetes increased glycation products (furosine and CML), whereas chronic kidney disease increased carbamylation products (homocitrulline) in the skin and aorta and in skin type I collagen. 2. Carbamylation of proteins precedes their glycation, with the former competitively inhibiting the latter protein modification.	Nicolas et al., 2019 [51]
Sprague–Dawley rats	Diabetes induced by streptozotocin (50 mg kg^−1^, i.p.)	1. Full-thickness excisional wounds increased AGE levels, interleukin (IL) 8 receptor A, leukotriene (LT) B4, and myeloperoxidase (MPO) in the skin wound edges more highly in the diabetic group.	Kang et al., 2021 [52]
KK-Ay/TaJcl mice (C57BL/6N background) and control C57BL/6j mice	Ages (10, 27, 40, and 50 weeks)	1. The body weight, blood glucose, skin thickness, transepidermal water loss (TEWL), AGE levels, and expression of RAGE, prostaglandin (PG) E_2_, tumor necrosis factor (TNF)-α, monocyte chemoattractant protein (MCP)-1, and C-C chemokine receptor (CCR) 2 were higher and the skin conductance and collagen expression were lower in the test groups compared to the age-matched control groups. 2. The age-dependent changes in these parameters were greater in the test groups than in the age-matched control groups.	Hiramoto et al., 2023 [53]
C57BL/6J mice	High-sugar feed	1. Changed toward a redder, yellower, and darker skin color. 2. Increased sebum secretion and lowered TEWL. 3. Increased skin AGE levels and decreased the expression of collagen type I, fibronectin 1, laminin-5, and tenascin C.	Li et al., 2024 [54]

**Table 2:** Clinical studies on the detection of AGEs in the skin and observation of glycation-mediated skin aging.

Model	Factors	Findings	References
Plasma samples from 30 living human subjects	Diabetes	CMA level of serum proteins was elevated in the diabetic group compared to the age-matched control group.	Odani et al., 2001 [55]
49 Japanese patients with diabetes	Diabetes	1. The high-frequency conductance (skin hydration) and skin surface lipid levels were lower in the group with high fasting plasma glucose (>110 mg dL^−1^). 2. The TEWL was slightly reduced in the group with high HbA1c levels (>5.8%).	Sakai et al., 2005 [56]
40 healthy Japanese women	Age	1. Cheek skin yellowness (b* value) and the AGE index, but not the melanin index, increased with age. 2. The b* value was correlated with the AGE index or the melanin index.	Ohshima et al., 2009 [57]
Group I (232 men) and group II (138 men) among 1263 participants enrolled in annual examinations	Age	1. Skin autofluorescence increased with age in group I and group II. 2. Participants with higher skin autofluorescence had lower grip strength (group I) and leg extension power (group II).	Momma et al., 2011 [58]
1441 human subjects with type I diabetes (726 with no retinopathy and 715 with nonproliferative retinopathy)	Diabetes	1. Skin autofluorescence in the log scale was correlated with mean HbA1c over time, age, smoking, skin tone, renal damage, and locational latitude.	Cleary et al., 2013 [59]
Five patient-matched skin biopsy specimens from chronic solar ultraviolet (UV) radiation-exposed and protected skin	UV exposure	1. Protein damage (e.g., AGEs) was higher in the UV-exposed skin. 2. DNA damage (e.g., 8-hydroxy deoxyguanosine) was higher in the UV-exposed skin. 3. Lipid peroxidation (e.g., 4-hydroxynonenal) was higher in the UV-exposed skin.	Mamalis et al., 2014 [60]
16 human subjects with diabetes (13 with type II diabetes, one with type I diabetes, and 2 without diabetes)	Wound	1. Expression of galectin-3, a potential receptor for AGEs, was reduced at the wound edge and in the wound bed where the AGE level was increased.	Pepe et al., 2014 [61]
A population-based cohort study involving 215 participants with type II diabetes	Diabetes	1. Higher skin autofluorescence levels were associated with delayed word recall and response inhibition. 2. Higher plasma levels of pentosidine, not CML or CEL, were associated with worse global cognitive functioning. 3. Associations did not differ between individuals with and without diabetes.	Spauwen et al., 2015 [62]
A total of 168 human subjects, including 82 subjects with type 2 diabetes	Diabetes	1. The autofluorescence intensity of fingertip skin and serum MG-H1, but not HbA1c, increased with the number of varied diabetic complications.	Yamanaka et al., 2016 [48]
A population-based prospective cohort study involving 2388 human subjects	Diabetes	1. Skin autofluorescence was higher in the diabetic group than in the non-diabetic group. 2. Serum 25-hydroxyvitamin D3 concentration was inversely associated with skin autofluorescence.	Chen et al., 2019 [63]
576 Japanese patients with primary open-angle glaucoma, exfoliation glaucoma, and non-glaucomatous controls	Glaucoma	1. AGE level (measured by fingertip skin autofluorescence) was higher in the exfoliation glaucoma group than in the primary open-angle glaucoma or control group. 2. Male sex, exfoliation glaucoma, and diabetes, but not age, visual acuity, intraocular pressure, glaucoma medications, lens status, and systemic hypertension, were associated with higher AGE levels.	Shirakami et al., 2020 [64]
2 healthy volunteers	UV exposure	1. Skin CML was increased by UV irradiation. 2. The CML level was higher in the paraffin-embedded skin specimens from sun-exposed areas than sun-protected areas of human subjects, and the increase was age-dependent.	Yoshinaga et al., 2012 [65]

**Table 3:** In vitro studies on the glycation of protein substrates and their inhibition by various materials.

Protein Substrates	Glycating Agents	Media and Additives	Reaction Conditions	Measurements	Test Materials	Positive Controls	References
Human serum albumin	Glucose (1.6 M)	Na-P buffer (50 mM, pH 7.2), 2 mM diethylenetriaminepentaacetic acid (DTPA)	37 °C, 7 days	Colorimetry with nitroblue tetrazolium (NBT) reaction.	NaBH_4_, DTPA	Aminoguanidine (AGD)	Hayashi et al., 2002 [69]
Bovine serum albumin (BSA) (0.149 mM)	Glucose (11 mM)	Physiological saline, 10 mM FeCl_2_	37 °C, 4 weeks	Spectrophotometry	Fucose- or rhamnose-rich oligo- and polysaccharides		Péterszegi et al., 2008 [70]
Bovine collagen type I	Ribose (10 mM)		Room temperature (RT), 1 month	Fluorimetry	Blueberry (*Vaccinium angustifolium*) extract	AGD	Pageon et al., 2008 [71]
BSA (1%)	Glucose 11 mM	10 mM FeCl_2_	37 °C, 4 weeks				Ravelojaona et al., 2009 [72]
α-Elastin (10 μg mL^−1^)	Ribose (0.2 M)	Phosphate-buffered saline (PBS)	37 °C, 1 week	Colorimetry (Elastase digestion)		AGD	Yoshinaga et al., 2012 [65]
Collagen (3 mg mL^−1^)	Glycolaldehyde (10 mM)	1 mM HCl, 0.02% NaN_3_, 1 mM DTPA	37 °C, 7 days	Fluorimetry	Gold nanoparticles	AGD	Kim et al., 2012 [73]
Bovine skin collagen type I (4–5 mg mL^−1^)	Ribose (10 mM)		RT, 3 weeks	Fluorimetry		AGD	Pageon et al., 2014 [74]
Collagen type I (1 mg mL^−1^)	Glucose (55 mM)	Dulbecco’s phosphate-buffered saline	45 °C, 3 weeks	Fluorimetry	*Manilkara* extract	AGD AGD AGD	Danoux et al., 2014 [75]
Bovine elastin (6 mg mL^−1^)	Glycolaldehyde (10 mM)		37 °C, 2 days		*Manilkara* and *Argania* extracts		
Albumin (6 mg mL^−1^)					*Argania* extract		
BSA (10 mg mL^−1^)	Glucose (0.5 M)	Phosphate buffer (0.1 M, pH 7.4), 0.02% NaN_3_	37 °C, 3 weeks	Fluorimetry	*Silybum marianum* flower extract, silibinin	AGD	Shin et al., 2015 [76]
					*Akebia quinata* fruit extract	AGD	Shin et al., 2015 [77]
Rat tail collagen type I (at (3–4 mg mL^−1^)	Sodium glyoxylate	0.5 N acetic acid, sodium cyanoborohydride	37 °C, 24 h	Fluorimetry		AGD	Pennacchi et al., 2015 [78]
BSA (20 mg mL^−1^)	Glyceraldehyde (20 mM)	K-phosphate buffer (0.1 M, pH 7.4), 1 mM DTPA	37 °C, 7 days	Sodium dodecyl sulfate-polyacrylamide gel electrophoresis (SDS-PAGE)			Han et al., 2016 [79]
BSA (20 mg mL^−1^)	Glucose (20 mM), glyoxal or methylglyoxal (29 μM)	PBS (0.2 M, pH 7.4), 1 mM NaN_3_	37 °C, 7 days	Fluorimetry	Rosmarinic acid, carnosine		Ou et al., 2017 [80]
BSA (10 mg mL^−1^)	Fructose (1.6 M)	Na-phosphate buffer (0.1 M, pH 7.4), 0.8% NaN_3_	37 °C, 7 days	Fluorimetry	*Cecropia pachystachya* leaf extract, quercetin	AGD	Fernandes et al., 2019 [81]
BSA (10 mg mL^−1^)	Ribose (0.5 M)	Na-phosphate buffer (50 mM, pH 7.4)	37 °C for 24 h	Fluorimetry	Mycosporine-like amino acids	AGD	Orfanoudaki et al., 2019 [82]
BSA (20 mg mL^−1^)	Glucose (0.5 M)	Phosphate buffer (0.1 M, pH 7.4), 0.02% NaN_3_	37 °C, 5 days	Fluorimetry	*Silybum marianum* flavonolignans		Drouet et al., 2019 [83]
BSA (10 mg mL^−1^)	Fructose (100 mM)	PBS (0.2 M, pH 7.2)	37 °C, 14 days	Fluorimetry	Black cumin *(Nigella sativa)* seed extract (rich in thymoquinone)	AGD	Li et al., 2020 [84]
Bovine collagen type I (1.5 mg mL^−1^)	Methylglyoxal (5 mM)	PBS, 10 mM NaN_3_	37 °C, 30 days				
BSA (1 mg mL^−1^)	Methylglyoxal (5 mM)	Phosphate buffer (pH 7.4), 150 mM NaCl	37 °C, 3 days	Fluorimetry	Alkaloids from *Ocotea paranapiacabensis*	AGD	Freitas et al., 2020 [85]
Collagen type I (2.5 mg mL^−1^)	Ribose (10 mM), glucose (200 mM)		RT, 7 days				
BSA (4 mg mL^−1^)	Methylglyoxal (20 mM)	PBS, 0.02% NaN_3_	37 °C, 7 days	Fluorimetry	Hazelnut (*Corylus avellana*) skin extracts, gallic acid	AGD	Spagnuolo et al., 2021 [86]
Collagen, elastin, BSA	Glyceraldehyde			Enzyme-linked immunosorbent assay (ELISA) for AGEs	Mycosporine-like amino acids	AGD	Waditee-Sirisattha and Kageyama, 2021 [87]
BSA (10 mg mL^−1^)	Glucose (0.5 M)	PBS, 1.5 mM phenylmethylsulfonyl fluoride (PMSF)	37 °C, 2 months	Fluorimetry			Kang et al., 2021 [52]
BSA (5 mg mL^−1^)	Methylglyoxal (25 mM)	Phosphate buffer (0.1 M, pH 8.0)	37 °C, 24 h	Colorimetry with NBT reaction	*Pholiota nameko* polysaccharides	AGD	Lin et al., 2021 [88]
BSA (10 mg mL^−1^)	Glucose (0.5 M)	Phosphate buffer (0.1 M, pH 7.4)	37 °C, 3 weeks	Fluorimetry for AGEs, colorimetry for protein carbonyls, and ELISA for CML	Methyl gallate	AGD	Shin et al., 2022 [89]
BSA (10 mg mL^−1^)	Glucose (0.5 M)	Phosphate buffer (0.1 M, pH 7.4)	37 °C, 28 days	Fluorimetry for AGEs, colorimetry for protein carbonyls, and ELISA for CML	*Cirsium japonicum* flower extract, apigenin, and chlorogenic acid	AGD	Yoon et al., 2022 [90]
BSA (10 mg mL^−1^)	Glycolaldehyde (10 mM)	Phosphate buffer (0.1 M, pH 7.4), 0.02% NaN_3_	37 °C, 5 days				
BSA (10 mg mL^−1^)	Glucose (0.5 M)	Phosphate buffer (0.1 M, pH 7.4)	37 °C, 4 weeks	Fluorimetry, ELISA for CML	Chlorella (*Parachlorella beijerinckii*) extract	AGD	Imai et al., 2022 [91]
BSA (2 mg mL^−1^)	Glyoxal (20 mM)		37 °C, 1 week				
Collagen (2 mg mL^−1^)	Glucose (100 mM)		37 °C, 4 weeks				
Collagen (1 mg mL^−1^)	Glyoxal (1 mM)		37 °C, 1 week				
BSA (50 mg mL^−1^)	Glucose (50 mM)	Na-phosphate buffer (0.2 M, pH 7.4), 0.02% NaN_3_	37 °C, 1 week	Fluorimetry	Vanillic acid	AGD	Alhadid et al., 2022 [92]
Collagen	Methylglyoxal, dehydroascorbic acid	PBS (0.1 M, pH 7.4)	37 °C, 7 days	Fluorimetry	Cranberry juice (rich in polyphenols)	AGD, alagebrium (ALT-711)	Chang et al., 2022 [93]
BSA (4 mg mL^−1^)	Methylglyoxal (20 mM)	PBS (pH 7.4)	37 °C, 168 h	Fluorimetry, dot blot for CML			Spagnuolo et al., 2023 [94]
BSA (10.35 mg mL^−1^)	Glucose (1 M)	PBS	50 °C, 6–7 days	Fluorimetry	*N*-Acetylhydroxyproline		Knoblich et al., 2024 [95]
BSA (10 mg mL^−1^)	Glyoxal (10 mM)	PBS (pH 7.4), 0.2% NaN_3_	37 °C, 7 days				Sultana et al., 2024 [96]

**Table 4:** Effects of glycation stress and various interventions on the pathological responses of cells in vitro. Upward (↑) and downward (↓) arrows indicate increases and decreases, respectively.

Model	Glycation Inducers	Induced Changes	Intervention	Outcomes	References
Reconstructed skin model	Ribose (10 mM)	1. CML, pentosidine (↑) 2. Integrin, collagen, procollagen, matrix metalloproteinases (MMPs) (↑)	AGD	1. CML, pentosidine (↓) 2. Integrin, collagen, procollagen (↓)	Pageon and Asselineau, 2005 [98]
A reconstructed skin model	CEL, CML, MG-H1, or pentosidine	1. Epidermal integrin α6 (↑) 2. Laminin-5 (↓) 3. Procollagen type I (↑) 4. MCP-1 (↑) 5. IL-6, MMP-1, MMP-3, vascular endothelial growth factor (VEGF) (↓)			Pageon et al., 2015 [99]
Reconstructed skin model	Ribose (10 mM)	1. Diameter of collagen lattice (↑) 2. Epidermal thickness (↑) 3. Dermal thickness (↓) 4. Dermal collagen aggregation (↑) 5. CML (↑) 6. Suprabasal integrin β1 (↑)	Blueberry extract, AGD	1. Diameter of collagen lattice (↓) 2. Epidermal thickness (↓) 3. Dermal thickness (↑) 4. Dermal collagen aggregation (↓) 5. CML (↓) 6. Suprabasal integrin β1 (↓)	Pageon et al., 2008 [71]
Human dermal fibroblasts (HDFs)	AGEs of BSA	1. Senescence-associated β-galactosidase (SA-β-gal)-positive cells (↑)	Fucose- or rhamnose-rich oligo- and polysaccharides	1. SA-β-gal-positive cells (↓)	Ravelojaona et al., 2009 [72]
HDFs	AGEs of BSA	1. MMP-2, MMP-9 (↑)	Fucose- or rhamnose-rich oligosaccharides	1. MMP-2, MMP-9 (↓)	Robert et al., 2010 [100]
Reconstructed skin model	Ribose (10 mM)	1. Dermal thickness (↓) 2. CML (↑) 3. Collagen type VII, procollagen type III, glycosaminoglycans (↑) 4. MMP-2 (↑)	AGD	1. Collagen type VII, procollagen type III, glycosaminoglycans (↓) 2. MMP-2 (↓)	Pageon et al., 2014 [74]
Reconstructed skin model (Mimeskin^TM^)	Ribose (0.5 M)	Diameter of collagen fibers (↓)	Davilla extract, AGD	Diameter of collagen fibers (↑)	Danoux et al., 2014 [75]
Dead human dermis	UV irradiation	1. Soluble fluorescent AGE levels (↑) 2. Soluble pentosidine-like AGE levels (↑) 3. Reactive oxygen species (ROS) production (↑)	*Argania* extract, AGD	1. Soluble fluorescent AGE levels (↓) 2. Soluble pentosidine-like AGE levels (↓) 3. ROS production (↓)	
HDFs	AGEs of BSA (100 μg mL^−1^)	1. ROS production (↑) 2. SA-β-gal-positive cells (↑)	*Akebia quinata* fruit extract	1. ROS production (↓) 2. SA-β-gal-positive cells (↓)	Shin et al., 2015 [77]
Endothelialized and innervated reconstructed skin model	Glyoxal (200–500 μM)	1. Increased CML (↑) 2. Number of capillaries (↓) 3. Platelet endothelial cell adhesion molecule (PECAM) 1, loricrin, filaggrin, and Krüppel-like factor (KLF) 4 (↓)	AGD, alagebrium (ALT-711)	1. Increased CML (↓) 2. Number of capillaries (↑) 3. PECAM-1, loricrin, filaggrin, and KLF-4 (↑)	Cadau et al., 2015 [101]
Reconstructed skin model	Sodium glyoxylate	1. Dermal thickness (↓) 2. CML expression (↑) 3. Cytokeratin (CK) 10 and CK-14 (↑) 4. E-cadherin and desmoglein (↑) 5. Gaps in the dermis (↑) 6. Collagen flattening/compression (↑)	AGD	1. Dermal thickness (↑) 2. CML expression (↓) 3. CK-10 and CK-14 proteins and mRNAs (↓)	Pennacchi et al., 2015 [78]
HDFs	UVB radiation with glyceraldehyde-induced AGEs of BSA (100 μg mL^−1^)	1. ROS production (↑) 2. RAGE protein (↑) 3. MMP-1 protein (↑) 4. TNF-α, IL-1β, and IL-6 (↑) 5. Phosphorylation of extracellular signal-regulated kinase (ERK), p38 mitogen-activated protein kinase (MAPK), and c-Jun *N*-terminal kinase (JNK) (↑) 6. Nuclear factor (NF)-κB/p65 subunit nuclear translocation and inhibitor of NF-κB (IκBα) phosphorylation (↑)	Plantamajoside	1. ROS production (↓) 2. RAGE protein (↓) 3. MMP-1 protein (↓) 4. TNF-α, IL-1β, and IL-6 (↓) 5. Phosphorylation of ERK, p38-MAPK, and JNK (↓) 6. IκBα phosphorylation and p65 nuclear translocation (↓)	Han et al., 2016 [79]
HaCaT keratinocytes					
HaCaT keratinocytes	Glyoxal (5 mM)	1. Cell viability (↓) 2. Fluorescent AGE levels (↑) 3. Free fatty acids (↑)			Yokota and Tokudome, 2016 [97]
Reconstructed skin model	Glyoxal (2.5–10 mM)	1. Free fatty acids (↑) 2. TEWL (↑)			
Dead mouse skin	Glyoxal 50 mM	1. Skin color yellowness (↑) 2. TEWL (↑)			
HDFs	Methylglyoxal-modified collagen	1. Cell viability (↓) 2. Apoptosis (↑) 3. Endoplasmic reticulum stress (↑) 4. Oxidative stress (↑)	*N*-Acetylcysteine	1. Apoptosis (↓) 2. Oxidative stress (↓)	Nowotny et al., 2018 [50]
Reconstructed skin model	Ribose (10 mM)	1. Epidermal thickness (↑) 2. Collagen aggregates (↑) 3. Fibril and filament (↑) 4. Integrin β1, laminin-5, loricrin (↑) 5. Filaggrin (↓)			Balansin Rigon et al., 2018 [102]
Reconstructed skin model	Ribose (10 mM), glucose (200 mM)	1. Dermis thickness (↓)	Alkaloids from *Ocoteaparanapiacabensis*, AGD	1. Dermis thickness (↑)	Freitas et al., 2020 [85]
HaCaT keratinocytes, human foreskin fibroblasts, THP-1 cells	t-Butylhydroperoxide	1. SA-β-gal-positive cells (↑) 2. IL-6, IL-8, MMP-1, MMP-3 (↑)	Carnosine	1. CD36, RAGE in macrophages derived from THP-1 cells (↑) 2. Protein kinase B (AKT) 2 phosphorylation (↑) 3. Macrophage-mediated elimination of senescent skin cells (↑).	Li et al., 2020 [103]
Hs68 cells	Methylglyoxal (400 μM)	1. Cell viability (↓) 2. ROS generation (↑)	*Pholiota nameko* polysaccharides	1. Cell viability (↑) 2. ROS generation (↓)	Lin et al., 2021 [88]
HL-60 cells	AGEs of BSA (0.5 μg mL^−1^)	1. Neutrophil migration and cluster formation (↓)			Kang et al., 2021 [52]
HDFs	UVB radiation	1. Pentosidine, AGEs, methylglyoxal (↑) 2. Denatured collagen (↑) 3. MMPs 1, 3, and 9 (↑)	Hydrolyzed fish collagen (25% tripeptide)	1. Pentosidine, AGEs, methylglyoxal (↓) 2. Denatured collagen (↓) 3. Collagen expression (↑) 4. MMPs 1, 3, and 9 (↓)	Lee et al., 2022 [104]
RAW264.7 cells	Methylglyoxal (100–400 μM)	1. Cell viability (↓)	Vanillic acid	1. Cell viability (↑)	Alhadid et al., 2022 [92]
HDFs	Glucose-induced AGEs (0.5 mM)	1. MMP-1 mRNA (↑)	*Cirsium japonicum* flower extract	1. MMP-1 mRNA (↓)	Yoon et al., 2022 [90]
Hs68 Cells	CML (100 μg mL^−1^)	1. ROS production (↑) 2. RAGE protein (↑) 3. Collagen contents (↓)	Djulis (*Chenopodium formosanum*) extract	1. ROS generation (↓) 2. RAGE protein (↓) 3. Collagen contents (↑)	Lyu et al., 2022 [105]
HaCaT keratinocytes	UVA radiation	1. AGE levels (↑)	Sunflower sprout extract	1. AGE levels (↓)	Barua et al., 2022 [106]
Fibroblasts in collagen gels	Glyoxal (400, 1000 μM)	1. Contraction of collagen gel (↓) 2. Fluorescent AGE levels (↑) 3. CML-protein (↑) 4. CMA-protein (↑) 5. RAGE protein (↑) 6. IL-8 mRNA (↑)	Chlorella (*Parachlorella beijerinckii*) extract, AGD	1. Contraction of collagen gel (↑) 2. Fluorescent AGE levels (↓) 3. CML-protein (↓) 4. CMA-protein (↑) 5. RAGE protein (↓) 6. IL-8 mRNA (↓)	Imai et al., 2022 [91]
HDFs	Methylglyoxal (500 μM)	1. Autofluorescence (AGEs) (↑) 2. Cell density and proliferation (↓) 3. ROS production (↑)	Carnosine, resveratrol, oxyresveratrol, piceatannol	1. Autofluorescence (AGEs) (↑) 2. Cell density and proliferation (↓) 3. ROS production (↑)	Markiewicz et al., 2022 [107]
Full-thickness skin model (EpiDermFT)		1. Skin model diameter (↓) 2. Autofluorescence (AGEs) (↑) 3. CML (↑) 4. Eosin fluorescence (↓) 5. Epidermal thickness (↓) 6. Cell density and proliferation (↓) 7. Collagen density (↓)		1. Autofluorescence (AGEs) (↓) 2. CML (↓) 3. Eosin fluorescence (↑) 4. Epidermal thickness (↑) 5. Cell density and proliferation (↑) 6. Collagen density (↑)	
HDFs (formaldehyde-fixed)	Glyoxal (0.5%)	1. AGE levels (↑)	*Jasminum sambac* cell extract	1. AGE levels (↓)	Ceccacci et al., 2022 [108]
Macrophages derived from THP-1 cells	Methylglyoxal-modified BSA (300 μg mL^−1^)	1. Cell viability (↓) 2. ROS production (↑) 3. TNF-α and IL-1β (↑)	Hazelnut (*Corylus avellana*) skin extract (rich in polyphenols)	1. Cell viability (↑) 2. ROS production (↓) 3. TNF-α and IL-1β (↓)	Spagnuolo et al., 2023 [94]
HDFs	Methylglyoxal (400 μM)	1. Cell viability (↓) 2. SA-β-gal-positive cells (↑) 3. Collagen type I alpha 1 chain (COL1A1) (↓) 4. AKT, JNK, p38-MAPK, c-Jun, and NF-κB phosphorylation (↑) 5. MMP-1 (↑)	*Carica papaya* fruit extract, AGD	1. Cell viability (↑) 2. SA-β-gal-positive cells (↓) 3. COL1A1 (↑) 4. AKT, JNK, p38-MAPK, c-Jun, and NF-κB phosphorylation (↓) 5. MMP-1 (↓)	Wattanapitayakul et al., 2023 [109]
Fibroblasts in collagen gels	Glyoxal (600 μM)	1. Contraction of collagen gel (↓)	*N*-Acetylhydroxyproline	1. Contraction of collagen gel (↑)	Knoblich et al., 2024 [95]
HDFs	Methylglyoxal (500 μM)	1. AGE levels (↑) 2. RAGE protein (↑)	Supramolecular carnosine	1. AGE levels (↓) 2. RAGE protein (↓)	Bai et al., 2024 [110]
HDFs	H_2_O_2_ (25 μM)	1. SA-β-gal-positive cells (↑) 2. Superoxide dismutase (SOD), catalase (CAT) activity (↓) 3. p-NF-κB/NF-κB ratio (↑) 4. IL-1β, IL-6 (↑) 5. MMP-1, MMP-9 (↑) 6. AGE levels (↑)	K formulation (containing hyaluronan, collagen type I peptide)	1. SOD, CAT activity (↑) 2. Nuclear factor erythroid 2-related factor (NRF) 2 expression (↑) 3. p-NF-κB/NF-κB ratio (↓) 4. IL-1β, IL-6 (↓) 5. MMP-1, MMP-9 (↓) 6. AGE levels (↓)	Augello et al., 2024 [111]
HDFs, human epidermal keratinocytes (HEKs), HaCaT keratinocytes	Glyoxal-modified BSA (100 μg mL^−1^), UVB radiation	1. Cell viability (↓) 2. Nitric oxide and ROS production (↑) 3. TNF-α, IL-1β, IL-6, IL-8 (↑) 4. NF-κB/p65 phosphorylation 5. RAGE and cyclooxygenase (COX) 2 (↑) 6. COL1A and NAD-dependent deacetylase sirtuin-1 (SIRT1) (↓)			Sultana et al., 2024 [96]

**Table 5:** Effects of glycation and various interventions on the pathological responses of skin explants ex vivo. Upward (↑) and downward (↓) arrows indicate increases and decreases, respectively.

Model	Glycation Inducers	Induced Changes	Interventions	Outcomes	References
Human skin explants	Methylglyoxal (500 μM)	1. Fibrillin-1 protein (↓) 2. CML expression (↑)	Silybum marianum flower extract, AGD	1. Fibrillin-1 protein (↑) 2. CML expression (↓)	Shin et al., 2015 [76]
Human skin explants	Methylglyoxal (500 μM)	1. Fibrillin-1 protein (↓) 2. CML expression (↑)	Akebia quinata fruit extract, AGD	1. Fibrillin-1 protein (↑) 2. CML expression (↓)	Shin et al., 2015 [77]
Human skin explants	Methylglyoxal (500 μM)	1. Pentosidine expression (↑) 2. CML expression (↑)	Carnosine	1. Pentosidine expression (↓) 2. CML expression (↓)	Narda et al., 2018 [113]
Human skin explants	Methylglyoxal (500 μM)	1. Fibrillin-1 protein (↓) 2. CML expression (↑)	Methyl gallate	1. Fibrillin-1 protein (↑) 2. CML expression (↓)	Shin et al., 2022 [89]
Human skin explants	Methylglyoxal (500 μM)	1. CML expression (↑)	Dunaliella salina extract	1. RAGE (↓) 2. IL-6 and IL-8 (↓) 3. NRF2 (↑)	Havas et al., 2022 [114]
Human skin explants	Methylglyoxal (500 μM)	1. Fibrillin-1 protein (↓)	Jasminum sambac cell extract, AGD	1. Fibrillin-1 protein (↑)	Ceccacci et al., 2022 [108]
Human breast skin explants	Glycolaldehyde (25 mM)	1. Autofluorescence (↑)	Fructosamine 3-kinase, fructosyl-amino acid oxidase	1. Autofluorescence (↓)	De Decker et al., 2023 [115]
Hypertrophic scar tissue explants			Fructosamine 3-kinase	1. Elongation rate (↑)	
Skin explants	Glucose (1 M)	1. Autofluorescence (↑)	*N*-Acetylhydroxyproline	1. Autofluorescence (↓)	Knoblich et al., 2024 [95]

**Table 6:** Effects of glycation and various interventions on the pathological responses of the skin in experimental animals in vivo. Upward (↑) and downward (↓) arrows indicate increases and decreases, respectively.

Model	Glycation Inducers	Induced Changes	Interventions	Outcomes	References
Wistar rats	Streptozotocin (50 mg kg^−1^, i.p.)	1. Blood glucose (↑) 2. Glycosylated Hb (↑) 3. Skin collagen fluorescence (↑)	Rutin (1 g L^−1^) or AGD (1 g L^−1^) in drinking water	1. Skin collagen fluorescence (↓)	Odetti et al., 1990 [117]
Emory mice prone to age-related cataract	Age (6.5–22 months)	1. Plasma glucose (↑) 2. Tail tendon breakdown time (↑) 3. Dermatological lesions (↑)	Restricted diet with different compositions (high protein and low carbohydrates)	1. Lifespan (↑) 2. Cataract grade (↓) 3. Plasma glucose (↓) 4. Glycohemoglobin (↓) 5. Tail tendon breakdown time (↓) 6. Dermatological lesions (↓)	Taylor et al., 1995 [118]
Brown-Norway rats	Age (4–25 months)	1. CML and pentosidine in skin collagen (↑)	Caloric restriction	1. CML, pentosidine, and fluorescence in collagen (↓)	Cefalu et al., 1995 [26]
Fischer 344 rats,	Age (6–24 months)	1. Furosine and pentosidine in skin collagen (↑)	Diet restriction	1. Furosine and pentosidine in skin collagen (↓)	Sell, 1997 [119]
C57BL/6NNia mice	Age (1–26 months)				
Sprague–Dawley rats	Age (2–24 months)	1. Skin and aortic collagen-linked fluorescence (↑) 2. Glycated plasma protein and Hb (↓)	Diet restriction	1. Glycated plasma proteins and fluorescent products in skin collagen of younger rats (↓) 2. Did not affect glycated Hb or aortic collagen fluorescence.	Novelli et al., 1998 [120]
BALB/c mice	Glucose (10% in feed)	1. Blood glucose (-) 2. Fluorescent AGE levels in dorsal subcutaneous tissues (↑) 3. Calibers of arterial microvessels (↓) 4. Vascular lesion indices (↑)	AGD (0.25%) in drinking water	1. Calibers of arterial microvessels (↑) 2. Vascular lesion indices (↓)	Yamada and Ohkubo, 1999 [121]
Broiler breeder chicks	Age (8–92 weeks)	1. Skin pentosidine (↑)	Diet restriction (60% of control) and AGD (400 ppm) in feed	1. Skin pentosidine (↓)	Iqbal et al., 1999 [122]
C57 BL/6J mice	Galactose (50 mg kg^−1^, s.c.)	1. Serum AGE levels (↑) 2. Motor activity (↓) 3. Memory latency time (↓) 4. Memory error (↑) 5. Lymphocyte proliferation (↓) 6. IL-2 production (↓)	AGD (0.1%) in drinking water	1. Serum AGE levels (↑) 2. Motor activity (↑) 3. Memory latency time (↑) 4. Memory error (↓) 5. Lymphocyte proliferation (↑) 6. IL-2 production (↑)	Song et al., 1999 [123]
Fischer 344 rats	Age (3–26 months), diets with different carbohydrate sources	1. The source of dietary carbohydrates (cornstarch, sucrose, glucose, fructose, or a combination of glucose and fructose) had little effect on serum glycemic stress and AGE levels	Diet restriction	1. Serum glucose and glycated Hb (↓) 2. Pentosidine in the collagen of a tail tendon (not skin or trachea) (↓)	Lingelbach et al., 2000 [124]
Broiler breeder chicks	Allopurinol (10 mg kg^−1^, p.o.) or hemin (10 mg kg^−1^, p.o.)	1. Skin pentosidine (↑)	Diet restriction	1. Skin pentosidine (↓)	Klandorf et al., 2001 [125]
Fisher 334 rats	Age (6–24 months)	1. Plasma glucose (↑) 2. Tail tendon breakdown time (↑) 3. Glycation (furosine) and glycoxidation (pentosidine and CML) of skin collagen (↑)	AGD (1 g L^−1^) in drinking water	1. There were no effects except for marginal effects on tail tendon breakdown time	Sell et al., 2001 [126]
C57BL/6 mice	Age (1, 4, 8–10.5 months)	1. Skin furosine and pentosidine (↑) 2. Tail collagen fluorescence (↑) 3. Tail tendon breakdown time (↑)	Vitamins C and E, blueberry, and green tea extracts	1. (Green tea extract) tail collagen fluorescence (↓) 2. (Green tea extract) tail tendon breakdown time (↓)	Rutter et al., 2003 [127]
Sprague–Dawley rats	Streptozotocin (45 mg kg^−1^)	1. Crosslinking of skin collagen (↑) 2. Acid solubility of tail collagen (↓)	*N*-phenacylthiazolium and *N*-phenylacy-4,5-dimethylthiazolium halides, pyridoxamine	1. There were no effects	Yang et al., 2003 [37]
Lewis rats	Streptozotocin (65 mg kg^−1^, i.v.)	1. Red blood cell-immunoglobulin (Ig) G crosslink (↑) 2. Tail tendon collagen crosslink (↑)	ALT-711 (10 mg kg^−1^, oral)	1. Red blood cell-IgG crosslink (↓) 2. Tail tendon collagen crosslink (↓)	Vasan et al., 2003 [36]
Fischer 344 rats	Aged 24 months		ALT-711 (5%, topical)	1. Skin elasticity (↑) 2. Skin hydration (↑)	
Squirrel and rhesus monkeys	Age	1. Rate of furosine formation in skin collagen (↑?) 2. Rate of pentosidine formation in skin collagen (↑?) 3. No change for CML	Diet restriction (70% of control)	1. Rate of furosine formation in skin collagen of rhesus monkeys (↓) 2. No change in pentosidine or CML level	Sell et al., 2003 [128]
Nude mice	Galactose (1000 mg kg^−1^, s.c.)	1. Serum AGE levels (↑) 2. Skin SOD activity (↓) 3. Skin MDA level (↑) 4. Dermal thickness (↓) 5. Skin collagen (↓) 6. Skin CD31-positive vessels density (↓) 7. Skin VEGF expression (↓)	Adipose-derived stem cells (1 × 10^6^ per injection)	1. Serum AGE levels (↓) 2. Skin SOD activity (↑) 3. Skin MDA level (↓) 4. Dermal thickness ↑) 5. Skin collagen (↑) 6. Skin CD31-positive vessels density (↑) 7. Skin VEGF expression (↑)	Zhang et al., 2014 [129], Wang et al., 2016 [130]
Cormorants	Age (1–5 years)	1. Hydroxyproline (↑) 2. Pentosidine (↑)			Dorr et al., 2017 [131]
ICR mice	Galactose (120 mg kg^−1^, i.p.)	1. Skin AGE levels (↑) 2. Skin collagen type I, III (↓) 3. Skin hyaluronan (↓) 4. Skin H_2_O_2_ (↑)	*Lactobacillus fermentum* CQPC04-fermented soy milk (10 mL kg^−1^)	1. Skin AGE levels (↓) 2. Skin collagen type I, III (↑) 3. Skin hyaluronan (↑) 4. Skin H_2_O_2_ (↓)	Zhou et al., 2021 [132]
DBA/2CrSlc mice	UV radiation	1. Ear skin Cu/Zn-SOD expression and total SOD activity (↓) 2. Ear skin CAT expression and activity (↓)	Mycosporine-like amino acid-containing emulsions (0.25 μmol g^−1^) (50 mg per ear, topical)	1. Skin Cu/Zn-SOD expression and total SOD activity (↑) 2. Skin CAT expression and activity (↑) 3. No changes in AGE levels of ear skin	Waditee-Sirisattha and Kageyama, 2021 [87]
Sprague–Dawley rats	Streptozotocin (35 mg kg^−1^, i.p.)	1. Serum fructosamine and HbA1c (↑) 2. AGE levels in the kidneys and tail skin (↑)	Vanillic acid (1.5, 4.5, or 15 mg kg^−1^, i.p.)	1. AGE levels in the kidneys and tail skin (↓)	Alhadid et al., 2022 [92]
HR-1 mice	UVB radiation	1. Wrinkle area, length, and depth (↑) 2. Skin and epidermal thickness (↑) 3. Skin moisture (↓) 4. Skin collagen and hyaluronan (↓) 5. Skin levels of AGEs and RAGE (↑)	*Schizonepeta tenuifolia* extract (containing rosmarinic acid)	1. Wrinkle area, length, and depth (↓) 2. Skin and epidermal thickness (↓) 3. Skin moisture (↑) 4. Skin collagen and hyaluronan (↑) 5. Skin levels of AGEs and RAGE (↓)	Gu et al., 2023 [133]
C57BL/6 mice	UV radiation	1. Skin melanin (↑) 2. Skin ROS (↑) 3. Skin AGE levels (↑)	Idebenone-loaded nanoparticles	1. Skin melanin (↓) 2. Skin ROS (↓) 3. Skin AGE levels (↓)	Xie et al., 2023 [134]
Hairless mice	Streptozotocin (45 mg kg^−1^, i.p.)	1. Skin wrinkle index (↑) 2. Skin hydration (↓) 3. Skin elasticity (↓) 4. Blood and skin levels of AGEs, CML, RAGE (↑) 5. Skin collagen and hyaluronan (↓) 6. Skin MMP-9 (↑) 7. Skin SOD and CAT activity (↓) 8. Skin TNF-α, IL-6 (↑)	Goji berry, fig, and Korean mint extracts)	1. Skin wrinkle index (↓) 2. Skin hydration (↑) 3. Skin elasticity (↑) 4. Blood and skin levels of AGEs and skin CML (↓) 5. Skin collagen (↑) 6. Skin IL-6 (↓)	Yoo et al., 2023 [135]
Swiss albino mice	Galactose (500 mg kg^−1^, i.g.)	1. Skin TNF-α, IL-1β, and malondialdehyde (MDA) (↑) 2. Skin glutathione peroxidase (GPX) (↓) 3. Skin collagen type I, III (↓)	Dapagliflozin (1 mg kg^−1^)	1. TNF-α, IL-1β, and MDA (↓) 2. Skin GPX (↑) 3. Skin collagen type I, III (↑)	Shihab et al., 2024 [136]

**Table 7:** Clinical studies on the effects of various interventions on the glycation levels and skin aging parameters in human subjects.

Study Format or Size	Test Materials	Treatments	Outcomes	References
A randomized, placebo-controlled study involving 22 subjects with type 2 diabetes	A dietary supplement containing 400 mg of vitamin E and 500 mg of vitamin C	The oral supplement was taken daily for a year.	1. Neither the treatment nor placebo group had significant changes in AGE level.	Konen et al., 2000 [137]
A double-blinded, placebo-controlled trial involving 40 women	A cream containing 10% green tea extract, an oral supplement (300 mg) containing green tea extract	The cream was applied to the face and arms, and the oral supplement was taken twice a day for 8 weeks.	1. No significant effects on the scoring of skin parameters by a physician and patients. 2. Histologically improved elastic tissue content of treated specimens.	Chiu et al., 2005 [138]
A single-center study enrolling 20 women with type II diabetes aged >50	A topical product formulation containing blueberry extract and C-xyloside	Subjects used the product on their face, hands, and inner forearms twice daily for 12 weeks.	1. Increased skin thickness and hydration. 2. Improved fine lines, firmness, radiance, skin tone, smoothness, creping, and overall appearance.	Draelos et al., 2009 [139]
A randomized, double-blinded, controlled study involving 42 subjects	A capsule containing carnosine, *N*-acetylcysteine, histidine, vitamin E, pantethine, methionine, and zinc picolinate	The capsule was taken orally every second day for 3 months, followed by a 1-month supplementation-free period.	1. Improved skin appearance, fine lines, and shiny look. 2. Improved the skin surface parameters, contrast, and circular roughness.	Babizhayev et al., 2012 [140]
21 adult volunteers	A cream containing 3% *Argania* plant extract, α-tocopheryl acetate, rutin, and ferulic acid	The cream was applied to the skin for 2 weeks.	1. Decreased the UVA-induced chemiluminescence of the skin, a measure of free radical production.	Danoux et al., 2014 [75]
A single-center, randomized controlled trial on 57 post-menopausal women	An equol supplement containing 98% *S*-equol, 2% daidzein, 0.2% glycitein, and 0.1% genistein	The supplement (10 mg) was taken orally for 3 months.	1. Improved climactic symptoms without effects on metabolic or aging-related biomarkers, including AGEs. 2. In certain populations (internal equol producers supplemented with external equol), AGE levels and visceral fat area were reduced.	Yoshikata et al., 2021 [141]
Twenty-two healthy women	An ampule containing hydrolyzed fish collagen (25% tripeptide)	The ampule was applied to the entire face twice daily for 4 weeks.	1. Improved periorbital and glabellar skin wrinkles, skin surface elasticity, and dermal density. 2. Reduced skin AGE levels.	Lee et al., 2022 [104]
A randomized, double-blinded, split-face, placebo-controlled trial on 25 female volunteers	A gel cream containing *Dunaliella salina* extract (rich in colorless carotenoids, such as phytoene and phytofluene)	One side of the face received the active product, and the other received the placebo for 56 days.	1. Reduced skin AGE levels. 2. Reduced histamine-stimulated microcircular blood flow. 3. Reduced periocular wrinkles and red spots.	Havas et al., 2022 [114]
Female subjects aged from 50 to 65	A lotion containing 0.025% *Cirsium japonicum* flower extract	Topically applied to the face twice a day for 8 weeks.	1. Decreased depth and volume of wrinkles. 2. Reduced maximum depth of the biggest wrinkle but not the total wrinkle area. 3. Increased skin elasticity.	Yoon et al., 2022 [90]
A double-blinded, randomized, controlled, single-center case study on 28 female subjects.	A serum containing sunflower sprout extract	The product was applied alone or in combination with a moisturizer lotion twice daily for 7 days.	1. Improved facial conditions, such as radiance, smoothness (tactile), fine lines of crow’s feet, and overall eye appearance.	Barua et al., 2022 [106]
A randomized, double-blinded, placebo-controlled trial on 31 individuals	Fish-derived collagen peptides (rich in prolyl-hydroxyproline and hydroxyprolyl-glycine)	Ingested collagen peptide (5 g) or placebo (maltodextrin) daily for 12 weeks.	1. Improved skin AGE levels and insulin resistance index (homeostasis model assessment ratio).	Koizumi et al., 2023 [142]
A split-face, placebo-controlled study involving 32 volunteers	An essence containing 0.5%, 1%, or 2% supramolecular carnosine	Topically applied to the face for 28 days.	1. Reduced brown spots. 2. Increased lightness (L* value) and individual topology angle (ITA^o^) and decreased melanin index of the facial skin.	Bai et al., 2024 [110]
Single-blinded clinical trials with 9 and 34 participants	A serum that claims to be anti-aging	Topically applied to the face for 7 or 56 days.	1. Reduced skin AGE levels. 2. Improved skin brightness and hydration, but not skin texture (elasticity, firmness).	Zhang et al., 2025 [143]
A randomized, double-blinded, placebo-controlled trial on 104 female participants	A capsule containing 300 mg of rosemary extract, 2 μg of biotin, and 0.45 mg of zinc gluconate	Subjects were instructed to take two capsules three times daily for weeks 1 to 4, two times daily for weeks 5 to 8, and one capsule two times daily for weeks 9 to 12.	1. Decreased the levels of 4-hydroxynonenal-protein adducts in the skin compared to both baseline values and the placebo group at 12 weeks. 2. Reduced skin AGE levels compared to both baseline values and placebo controls at 12 weeks.	Guiotto et al., 2025 [144]

## Data Availability

The original contributions presented in this study are included in the article, and further inquiries can be directed to the corresponding author.

## Abbreviations

AGD	aminoguanidine
AGE	advanced glycation end-product
AKT	protein kinase B; AKT
BSA	bovine serum albumin
CAT	catalase
CCR	C-C chemokine receptor
CEL	*N*^ε^-(carboxyethyl)lysine
CK	cytokeratin
CMA	*N*^ω^-(carboxymethyl)arginine
CML	*N*^ε^-(carboxymethyl)lysine
COL	collagen
COX	cyclooxygenase
DTPA	diethylenetriaminepentaacetic acid
ELISA	enzyme-linked immunosorbent assay
ERK	extracellular signal-regulated kinase
GPX	glutathione peroxidase
Hb	hemoglobin
HDF	human dermal fibroblast
HEK	human epidermal keratinocyte
HUVEC	human umbilical vein endothelial cell
Ig	immunoglobulin
IκB	inhibitor of NF-κB
IL	interleukin
ITA^o^	individual topology angle
JNK	c-Jun N-terminal kinase
KLF	krüppel-like factor
LT	leukotriene
MAPK	mitogen-activated protein kinase
MCP	monocyte chemoattractant protein
MDA	malondialdehyde
MG-H1	methylglyoxal-derived hydroimidazolone 1; *N*^δ^-(5-hydro-5-methyl-4-imidazolon-2-yl)-ornithine
MMP	matrix metalloproteinase
MPO	myeloperoxidase
NBT	nitroblue tetrazolium
NF	nuclear factor
NRF	nuclear factor erythroid 2-related factor
PBS	phosphate-buffered saline
PECAM	platelet endothelial cell adhesion molecule
PG	prostaglandin
PMSF	phenylmethylsulfonyl fluoride
RAGE	receptor for advanced glycation end-products
ROS	reactive oxygen species
RT	room temperature
SA-β-gal	senescence-associated β-galactosidase
SDS-PAGE	sodium dodecyl sulfate-polyacrylamide gel electrophoresis
SIRT	NAD-dependent deacetylase sirtuin
SOD	superoxide dismutase
TEWL	transepidermal water loss
TNF	tumor necrosis factor
UV	ultraviolet
VEGF	vascular endothelial growth factor
